# Management of Rectal Cancer and Liver Metastatic Disease: Which Comes First?

**DOI:** 10.1155/2012/196908

**Published:** 2012-06-20

**Authors:** Georgios Tsoulfas, Manousos-Georgios Pramateftakis

**Affiliations:** ^1^1st Surgical Department, Aristotle University of Thessaloniki, Papageorgiou General Hospital, 56429 Thessaloniki, Greece; ^2^4th Surgical Department, Aristotle University of Thessaloniki, Papanikolaou General Hospital, 57010 Thessaloniki, Greece

## Abstract

In the last few decades there have been significant changes in the approach to rectal cancer management. A multimodality approach and advanced surgical techniques have led to an expansion of the treatment of metastatic disease, with improved survival. Hepatic metastases are present at one point or another in about 50% of patients with colorectal cancer, with surgical resection being the only chance for cure. As the use of multimodality treatment has allowed the tackling of more complicated cases, one of the main questions that remain unanswered is the management of patients with synchronous rectal cancer and hepatic metastatic lesions. The question is one of priority, with all possible options being explored. Specifically, these include the simultaneous rectal cancer and hepatic metastases resection, the rectal cancer followed by chemotherapy and then by the liver resection, and finally the “liver-first” option. This paper will review the three treatment options and attempt to dissect the indications for each. In addition, the role of laparoscopy in the synchronous resection of rectal cancer and hepatic metastases will be reviewed in order to identify future trends.

## 1. Introduction

Rectal cancer is the fourth most commonly diagnosed malignancy in the United States, with approximately one-third of all colorectal malignancies arising in the rectum, considered as the last 15 cm of the large bowel. Of the 150,000 new cases of colorectal cancer (CRC) diagnosed in the United States every year, about 50% develop hepatic metastases during the course of their disease, with 20–25% of these presenting with synchronous liver metastases [1–3]. This is even more important if we consider that in about a one-third of the patients with synchronous or metachronous liver metastases, the liver is the only site of metastatic disease, meaning that around 15,000 patients per year are candidates for therapy of these lesions [4]. 

 Just as important as the extent of the disease is the fact that the only potential therapy for cure in patients with CRC and hepatic metastatic disease is surgery [5]. Even though median survival of patients with untreated metastatic CRC is around 6 months to a year, advances in adjuvant treatment after the colorectal resection have shown the potential of a decrease in the number of metastatic cases [6]. Agents such as oxaliplatin, and newer targeted-therapy ones such as cetuximab and bevacizumab, have led to improved response rates and survival [7–9]. Even so, the two-year survival is limited to 40% at best, thus reiterating the primacy of surgery as part of the multimodal approach [10]. Limitations remain as only about 10–20% of patients with liver metastatic disease are candidates for surgical resection at presentation [11]. However, another 15–30% of previously considered unresectable patients can be converted and (despite the absence of randomized controlled trials) the majority of evidence supports a significant survival benefit with surgical resection, with overall 5-year survival rates after hepatic resection with curative intent ranging from 35 to 55% [12–16].

 All of this progress in our understanding and management of patients with CRC and hepatic metastatic disease, not unexpectedly, has led to more questions. A central one is the timing and sequence of therapeutic interventions in patients presenting with synchronous CRC and hepatic metastases. The classical approach has been to resect the CRC, continue with chemotherapy, and then proceed to the liver, provided that the patient is coping with the treatments and the hepatic disease burden is manageable. Improvement in surgical and anesthesia techniques and the accumulation of experience have allowed qualified surgical teams to proceed with the simultaneous resection of both the CRC and the hepatic metastases in selected patients. The realization that the liver metastases are actually what defines the prognosis of the patients, and because complications in rectal surgery are not uncommon after chemoradiation and can thus delay the start of appropriate metastatic therapy, the “liver-first approach” has been proposed in patients with locally advanced rectal cancer and synchronous liver metastases. This paper will attempt to dissect the different types of approaches and identify the patients that would be most served by each one. Finally, the role of laparoscopy will also be reviewed to identify future directions in the management of stage IV rectal cancer.

## 2. Chemotherapy and Resectability

 There is a new paradigm in what is considered resectable liver metastatic disease, as previous standards having to do with the disease burden (how many lesions, location) have been replaced by newer ones that place the focus on what remains behind. Specifically, in order for hepatic metastatic lesions to be considered resectable it is important to be able to achieve a negative resection margin and to leave behind at least two contiguous segments with adequate size and function to avoid hepatic insufficiency after resection. The adequacy of the size of the liver remnant is dependent on its quality, which can certainly be affected by the preoperative chemotherapy. 

 This brings into the forefront the question of the ideal timing of surgery in patients with resectable disease. Preoperative chemotherapy used as neoadjuvant therapy offers the advantage of earlier treatment of micrometastatic disease and possible improved containment, as well as evaluation of tumor responsiveness, which is information that can also help shape future therapy [17]. Some argue that the presence of the whole liver may mean increased tolerability to the side effects of chemotherapy, compared to the hepatic remnant after resection. Tumor responsiveness can help determine future chemotherapy, as well as identify those patients with the best chance for resection, since progression while on chemotherapy is a bad prognostic factor [18, 19]. There are, however, complications which include chemotherapy-induced nonalcoholic steatohepatitis (NASH), steatosis, centrilobular necrosis and sinusoidal changes [20, 21]. More importantly, preoperative chemotherapy, due to the liver injury, can delay the resection or even simply make it very difficult to identify the lesion as a complete response does not necessarily mean that all cancer cells have been eradicated [22]. The advantages and disadvantages of preoperative chemotherapy mentioned above make it even harder to identify what the best sequence of therapies should be in patients presenting with simultaneous rectal lesions and resectable liver metastases. Even an expert consensus statement in 2006 failed to provide clear guidelines, stating that “either stages or simultaneous resections of the primary tumor and liver metastases can be considered depending on variable factors…” [23]. Even so, three main approaches (the classical staged one and the more novel synchronous and “liver-first” ones) have been identified and it is essential to understand the type of patient that each one is applicable for.

## 3. Simultaneous versus Staged Primary and Hepatic Metastases Resection

 The classical approach in dealing with synchronous rectal cancer liver metastases has been the staged one, where colectomy is followed by chemotherapy and then by liver resection, provided that the disease passes the “test of time” [24–26]. Advocates of this approach believe that it allows the full metastatic load of the disease to be revealed, as well as the biological behavior or “aggressiveness” of the tumor. An added argument is the potential for increased morbidity and mortality from the combination of two major operations. However, several studies have shown that the synchronous colorectal resection does not lead to increased morbidity or mortality when combined with partial hepatectomy [27–30]. There are two caveats here; the first one is the fact that most studies refer to colorectal cancer as a whole and not just rectal cancer. The importance of this is that rectal procedures are technically more challenging than colon procedures, with a higher risk of morbidity and mortality. Despite that, a study (possibly the only one identified in the literature) looking at synchronous rectal and hepatic resection of rectal metastatic disease from the Mayo Clinic showed that combined rectal and hepatic resection is safe [31]. They reported overall survival at 1, 2, and 5 years of 88%, 72%, and 32%, respectively, as well as disease-free survival from local recurrence at 1, 2, and 5 years of 92%, 86%, and 80%, numbers that were comparable to those undergoing a staged procedure. Similar data were presented in another published study from the Memorial Sloan-Kettering Cancer Center, which reported prospectively on 240 patients undergoing synchronous resection of a primary colorectal carcinoma [32]. In that study rectal cancers were 38% of the patient population, but in the simultaneously resected population most hepatic resections were not of the major type.

 The other caveat is that most studies comparing simultaneous and staged colorectal and hepatic resections are retrospective and, more importantly, patients undergoing the simultaneous procedure had fewer, smaller, and more often unilobar synchronous colorectal liver metastases [33]. These concerns have led to the recommendation that simultaneous procedures should only be pursued when they involve minor hepatic resections, while major hepatectomies should only occur in very carefully selected cases and by an experienced hepatobiliary team. Additionally, simultaneous hepatic resections should not be performed as part of an exploration for an emergent colorectal resection for bleeding or perforation or obstruction, because apart from the severely increased morbidity, they can also lead to a higher chance of distant metastases [34]. Similarly, if there is chronic significant liver disease, or the possibility of a small liver remnant, simultaneous resections should not be performed to avoid the risk of postoperative hepatic insufficiency.

## 4. The “Liver-First Approach”

 This approach is the latest and one that is most suited for patients with advanced rectal cancer. Approximately, 30% of patients with locally advanced rectal cancer have synchronous liver metastases. The locally advanced rectal disease is usually treated with a long course of chemoradiation of about five weeks and with at least six weeks going by before the patient can be operated upon. The result is that, and provided that there are no chemoradiation complications, three months will have passed before the liver disease is actually addressed. This is made harder by the high frequency of complications, which push any therapy for the liver disease even further down the road, as well as the fact that the liver metastatic disease is the one ultimately affecting the prognosis.

 The “liver-first” or “reverse” approach consists of preoperative chemotherapy, followed by resection of the hepatic metastatic disease, and then by resection of the rectal primary at a second operation. The ideal patient is one with advanced synchronous liver metastatic disease and a rectal cancer [35]. The rationale for this approach is based on the fact that complications such as bleeding, obstruction, or perforation are rare in patients with stage IV colorectal cancer, as well as the fact that treatment of the metastatic disease is not delayed by the local therapy for the primary tumor [36, 37]. An increasing number of studies have examined this approach, with one of the earliest ones by Mentha and colleagues demonstrating the safety of this strategy with morbidity and mortality rates of 19% and 0%, respectively, and an overall 3-year survival of 83% [38]. Another study comparing all 3 strategies, revealed similar results with morbidity and mortality of 31% and 4%, respectively, and a 3-year overall survival of 79% [39]. Most importantly, that study showed that the classic, combined, or reverse surgical strategies in patients with synchronous colorectal lesions and hepatic metastases are associated with similar outcomes [39]. The “liver-first” or reverse approach is best suited for patients with advanced hepatic metastases and an asymptomatic primary.

## 5. The Role of Laparoscopy

We have seen that the optimal strategy for managing resectable synchronous colorectal liver metastases is being refined over time. Although the guidelines have been to perform the colorectal cancer and the liver resection separately because of potentially increased mortality, this has been changed in several cases to one of synchronous resection for patients with limited hepatic metastatic disease [40–42]. Similar reluctance was observed in the case of stage IV rectal cancer specifically, because of the fear of increased risk of morbidity and anastomotic leakage [43]. The institution of a concerted approach by colorectal and hepatobiliary surgeons has led to the successful application of simultaneous resection, even in the case of major hepatic resections [44].

 Achieving a solid oncologic outcome in a safe manner further encouraged surgeons to the use of laparoscopic total mesorectal excision (TME) for stage IV rectal cancer combined with open or laparoscopic hepatic resection. One of the largest series for stage IV rectal cancer from Beaujon Hospital included 10 patients undergoing laparoscopic mesorectal excision, of which 3 underwent a simultaneous laparoscopic resection for the hepatic neoplasm and the other seven an open resection through a small incision [45]. In this pilot study it is suggested that laparoscopic rectal resection with synchronous resection of hepatic disease is possible with acceptable, low morbidity and a short hospital stay. Others have used a hybrid procedure, with the use of hand-assisted laparoscopic surgery for minor resections, whereas pure laparoscopic rectal and hepatic resections for stage IV rectal cancer have mainly appeared as case reports [46, 47].

 These reports show that technical progress and an improving learning curve can play a critical role in the more widespread use of laparoscopic surgery for the simultaneous resection of stage IV rectal cancer. However, we need to be mindful of the fact that this procedure remains under evaluation, as the learning curve and the operative time are long, and no randomized controlled trials exist [48].

## 6. Conclusion

The optimal timing for surgical resection of synchronous colorectal liver metastasis and the primary tumor is a question that remains unanswered. The traditional approach of a staged approach with initial resection of the colorectal tumor, followed by chemotherapy and then by hepatic resection 2 to 3 months later, has been based on the argument that it is physiologically less stressful for the patient compared to the combined procedure. This becomes even more important in the case of rectal cancer, where resection of the rectal primary is a significantly more challenging procedure by itself with well-established morbidity. Advances in surgical and perioperative care have changed this mindset, with the result being that the simultaneous resection of the colorectal primary and the hepatic metastases is increasingly gaining ground, as it results in morbidity, mortality, and length of stay comparable to the staged resection, while at the same time leading to earlier completion of the surgical therapy and the ability to start adjuvant therapy. With increasing experience it has also been possible to better define the subset of patients that would benefit the most from this approach, which includes those patients that warrant a limited hepatic resection.

 Further advances led to the institution of the “liver-first” approach, based on the belief that in patients with stage IV rectal cancer, the factor that defines survival is the ability to deal with the hepatic metastatic disease. In selected patients, where the primary rectal cancer is not a threat for bleeding, obstruction, or perforation, there is the option of addressing the hepatic disease first before it progresses too far. Furthermore, laparoscopic surgery with the rapid progress that it is experiencing is playing a role even in the cases of simultaneous resection. From the above discussion it becomes evident that trying to answer the question of timing of surgery for patients with colorectal primary and hepatic metastatic disease allows us to see how far we can push the envelope in providing sound surgical treatment for these patients.
